# Supplementary material for: From sequence to enzyme mechanism using multi-label machine learning
Source: BMC Bioinformatics. 2014 May 19;15:150. doi: 10.1186/1471-2105-15-150 (PMC4229970; doi:10.1186/1471-2105-15-150)
Supplement: Additional file 2 — Java code of ml2db. Additional file ml2db_code.tar.gz contains the Java source code to run the multi-label machine learning experiments and save the results to database. The code’s Javadoc is included. [file 1471-2105-15-150-S2.zip › additional file 2/ml2db/ml2db/doc/index-files/index-1.html]

A-Index


JavaScript is disabled on your browser.


- Overview
- Package
- Class
- Use
- Tree
- Deprecated
- Index
- Help

- Prev Letter
- Next Letter

- Frames
- No Frames

- All Classes

A B C D E F G H I J K L M N O P Q R S T U V W X 


## A

ActiveLearning - Class in main.ml2db.al
:   Executes pool-based active (machine) learning for nearest neighbours.

ActiveLearning(ExperimentDetails, int, String) - Constructor for class main.ml2db.al.ActiveLearning


ActiveLearning(ExperimentDetails, int, String, int) - Constructor for class main.ml2db.al.ActiveLearning


ActiveLearningDatabaseTest - Class in test.ml2db.al
:   Class

ActiveLearningDatabaseTest() - Constructor for class test.ml2db.al.ActiveLearningDatabaseTest


ActiveLearningTest - Class in test.ml2db.al
:   Class

ActiveLearningTest() - Constructor for class test.ml2db.al.ActiveLearningTest


add(String) - Method in class main.ml2db.graphviz.GraphViz
:   Adds a string to the graph's source (without newline).

addADistance(String, String, Double) - Method in class main.ml2db.al.utility.globaldistance.DistancesStorage


addAll(IdMultiLabelInstances) - Method in class main.ml2db.mulan.mulanarff.IdMultiLabelInstances
:   Instances must come from already compatible datasets!

addAll(MapOfMaps) - Method in class main.ml2db.weka.InstAttValueMap
:   Sets the type of attribute after adding data

addDistances(String, Double, int) - Method in class main.ml2db.al.utility.globaldistance.GlobalPositionStore
:   Add an instance as sparse instance attributes string: {attribute index
    attribute value, ...} eg.

addEvaluationResults(TreeMap<String, String>, Evaluation) - Method in class main.ml2db.mulan.learn.crosseval.EvaluationResultsTable
:   adds the evaluation results to the data map (table column 2 value map)

addInstance(IdInstance) - Method in class main.ml2db.mulan.mulanarff.IdMultiLabelInstances


addInstances(IdMultiLabelInstances) - Method in class main.ml2db.graphviz.GraphVizArff


addln() - Method in class main.ml2db.graphviz.GraphViz
:   Adds a newline to the graph's source.

addln(String) - Method in class main.ml2db.graphviz.GraphViz
:   Adds a string to the graph's source (with newline).

addNeighbour(Neighbourhood) - Method in class main.ml2db.al.utility.neighbours.Neighbourhood


addToInstanceNamesToAttributesStringMap(String) - Method in class main.ml2db.mulan.mulanarff.IdMultiLabelInstances


addToInstanceNamesToSparseAttrStringMap(String) - Method in class main.ml2db.mulan.mulanarff.IdMultiLabelInstances


AlDataSaver - Class in main.ml2db.al.database
:   Class

AlDataSaver(DbManager, String) - Constructor for class main.ml2db.al.database.AlDataSaver


AlDbCreator - Class in main.ml2db.al.database
:   Creates the tables to store Active Learning machine learning experiments and
    results.

AlDbCreator(DbManager) - Constructor for class main.ml2db.al.database.AlDbCreator


AlEvaluationTable - Class in main.ml2db.al.database
:   Class

AlEvaluationTable(DbManager) - Constructor for class main.ml2db.al.database.AlEvaluationTable


AlExperimenter - Class in main.ml2db.al.learning
:   Class

AlExperimenter(MultiLabelInstances, MultiLabelInstances, DbManagers, MulanLearner, String, ActiveLearning) - Constructor for class main.ml2db.al.learning.AlExperimenter


AlExperimenterTest - Class in test.ml2db.al
:   Class

AlExperimenterTest() - Constructor for class test.ml2db.al.AlExperimenterTest


AlExperimentsTable - Class in main.ml2db.al.database
:   Represents a table and table column names for storing details of an active
    learning experiment, such as: labelled and unlabelled arff file, algorithm
    used for evaluation, distance function used, utility function used etc.

AlExperimentsTable(DbManager) - Constructor for class main.ml2db.al.database.AlExperimentsTable


ALGORITHM - Static variable in class main.ml2db.al.database.AlExperimentsTable
:   the name for the algorithm table column

ALGORITHM - Static variable in class main.ml2db.mulan.learn.crosseval.EvaluationResultsTable
:   the name for the algorithm table column

algorithm() - Method in class main.ml2db.mulan.predict.PredictionsSaver


AllActiveLearningTests - Class in test.ml2db.alltests
:   Class

AllActiveLearningTests() - Constructor for class test.ml2db.alltests.AllActiveLearningTests


AllArffTests - Class in test.ml2db.alltests
:   Class

AllArffTests() - Constructor for class test.ml2db.alltests.AllArffTests


AllDataTests - Class in test.ml2db.alltests
:   Data harness for arff file generation and learning tests.

AllDataTests() - Constructor for class test.ml2db.alltests.AllDataTests


AllExternalPredictionsEvaluationTests - Class in test.ml2db.alltests
:   Class

AllExternalPredictionsEvaluationTests() - Constructor for class test.ml2db.alltests.AllExternalPredictionsEvaluationTests


AllGraphVizTests - Class in test.ml2db.alltests
:   Class

AllGraphVizTests() - Constructor for class test.ml2db.alltests.AllGraphVizTests


AllMulanArffTests - Class in test.ml2db.alltests
:   Class

AllMulanArffTests() - Constructor for class test.ml2db.alltests.AllMulanArffTests


AllMulanLearningTests - Class in test.ml2db.alltests
:   Class

AllMulanLearningTests() - Constructor for class test.ml2db.alltests.AllMulanLearningTests


AllMulanPredictionTests - Class in test.ml2db.alltests
:   Class

AllMulanPredictionTests() - Constructor for class test.ml2db.alltests.AllMulanPredictionTests


AllMulanTests - Class in test.ml2db.alltests
:   Class

AllMulanTests() - Constructor for class test.ml2db.alltests.AllMulanTests


AllPreliminaryTests - Class in test.ml2db.alltests
:   Class

AllPreliminaryTests() - Constructor for class test.ml2db.alltests.AllPreliminaryTests


AllTests - Class in test.ml2db.alltests
:   All tests Note: RUN AllDataTests FIRST to generate the database tables, some
    arff files, some data etc.

AllTests() - Constructor for class test.ml2db.alltests.AllTests


AllTrainTestsTests - Class in test.ml2db.alltests
:   Class

AllTrainTestsTests() - Constructor for class test.ml2db.alltests.AllTrainTestsTests


AlOutputManager - Class in main.ml2db.al.learning
:   Class

AlOutputManager(DbManager, String, Table, AlExperimenter) - Constructor for class main.ml2db.al.learning.AlOutputManager


AnnotationsReader - Class in main.ml2db.mulan.measuremetrics
:   Two sets of [instance, annotation] couples are taken as input, one containing
    "predictions" and another containing the "truth" .

AnnotationsReader(String, String, String, String) - Constructor for class main.ml2db.mulan.measuremetrics.AnnotationsReader


AnnotationsReaderTest - Class in test.ml2db.mulan.measuremetrics
:   Class

AnnotationsReaderTest() - Constructor for class test.ml2db.mulan.measuremetrics.AnnotationsReaderTest


Arff - Class in main.ml2db.weka
:   Creates an ARFF file: checks file properties, loads data from database, fills
    and writes to file the ARFF attributes, classes and instances, generates
    auxiliary files if needed.

Arff(String, DbManagers) - Constructor for class main.ml2db.weka.Arff


Arff(String, DbManagers, boolean) - Constructor for class main.ml2db.weka.Arff


ARFF\_DATA\_PROP - Static variable in class main.ml2db.weka.ArffProps
:   The full arff data file text taken the ARFF file.

ARFF\_DB\_PROPS - Static variable in class test.ml2db.dataharness.TestProjectParameters


arff\_EC\_Instance1 - Static variable in class test.ml2db.mulan.mulanarff.MulanArffTest


ARFF\_FILE\_PATH\_PROP - Static variable in class main.ml2db.weka.ArffProps
:   the file path where the ARFF file will be written, inclusive of file
    name.

ARFF\_FILES\_DIR - Static variable in class main.ml2db.ProjectParameters


ARFF\_FILES\_PATH - Static variable in class main.ml2db.ProjectParameters


ARFF\_FILES\_PATH - Static variable in class test.ml2db.dataharness.TestProjectParameters


ARFF\_ID\_COLUMN - Static variable in class main.ml2db.weka.ArffRecordsTable


ARFF\_PROPS\_NO\_XML - Static variable in class test.ml2db.dataharness.TestProjectParameters


ARFF\_PROPS\_NUMERIC\_ATT - Static variable in class test.ml2db.dataharness.TestProjectParameters


ARFF\_PROPS\_PATH - Static variable in class main.ml2db.ProjectParameters


ARFF\_PROPS\_PATH - Static variable in class test.ml2db.dataharness.TestProjectParameters


ARFF\_TABLE - Static variable in class main.ml2db.ProjectParameters
:   The name of the table to save the arff file details to

arffEmptyInstance - Static variable in class test.ml2db.dataharness.DataOne


arffEmptyInstance - Static variable in class test.ml2db.dataharness.DataTwo


ArffGeneratorForFilterTests - Class in test.ml2db.mulan.attributesfilter


ArffGeneratorForFilterTests() - Constructor for class test.ml2db.mulan.attributesfilter.ArffGeneratorForFilterTests


arffInstance1 - Static variable in class test.ml2db.dataharness.DataOne


arffInstance1 - Static variable in class test.ml2db.dataharness.DataTwo


arffInstance1 - Static variable in class test.ml2db.mulan.mulanarff.MulanArffTest


arffInstance1b - Static variable in class test.ml2db.dataharness.DataOne


arffInstance1b - Static variable in class test.ml2db.dataharness.DataTwo


arffInstance2 - Static variable in class test.ml2db.dataharness.DataOne


arffInstance2 - Static variable in class test.ml2db.dataharness.DataTwo


arffInstance2 - Static variable in class test.ml2db.mulan.mulanarff.MulanArffTest


arffInstance4 - Static variable in class test.ml2db.dataharness.DataOne


arffInstance4 - Static variable in class test.ml2db.dataharness.DataTwo


arffInstance4 - Static variable in class test.ml2db.mulan.mulanarff.MulanArffTest


arffInstanceAttributeVoid - Static variable in class test.ml2db.mulan.mulanarff.MulanArffTest


arffInstanceAttVoid - Static variable in class test.ml2db.dataharness.DataOne


arffInstanceAttVoid - Static variable in class test.ml2db.dataharness.DataTwo


arffInstanceClassAttributeVoid - Static variable in class test.ml2db.mulan.mulanarff.MulanArffTest


arffInstanceClassVoid - Static variable in class test.ml2db.dataharness.DataOne


arffInstanceClassVoid - Static variable in class test.ml2db.dataharness.DataTwo


arffInstanceClassVoid - Static variable in class test.ml2db.mulan.mulanarff.MulanArffTest


ArffProps - Class in main.ml2db.weka
:   A class containing the meta-data for a Weka ARFF file (or arff + xml files
    for Mulan ARFF) The data includes the path to an existing ARFF file, an
    optional data set name and description, and various statistics for the data
    (number of instances, class values, attributes)

ArffProps(String, String) - Constructor for class main.ml2db.weka.ArffProps
:   Xml path is there for compatibility with Mulan, can be left null or
    empty.

ArffProps(String, String, String) - Constructor for class main.ml2db.weka.ArffProps
:   A textual, human readable description for the data

ArffPropsFilesTest - Class in test.ml2db.dataharness
:   Test the ARFF properties files used in the test harness and their content.

ArffPropsFilesTest() - Constructor for class test.ml2db.dataharness.ArffPropsFilesTest


ArffPropsFromFile - Class in main.ml2db.weka
:   Contains the properties for a Weka ARFF file to be generated from database
    queries.

ArffPropsFromFile(String) - Constructor for class main.ml2db.weka.ArffPropsFromFile
:   Constructor loads the properties from .props properties file

ArffPropsOneTest - Class in test.ml2db.dataharness
:   Get the arff data properties from file

ArffPropsOneTest() - Constructor for class test.ml2db.dataharness.ArffPropsOneTest


ArffPropsQueriesOneTest - Class in test.ml2db.dataharness
:   Class

ArffPropsQueriesOneTest() - Constructor for class test.ml2db.dataharness.ArffPropsQueriesOneTest


ArffPropsQueriesTest - Class in test.ml2db.dataharness
:   Checks the results of the queries contained in the arff properties files

ArffPropsQueriesTest() - Constructor for class test.ml2db.dataharness.ArffPropsQueriesTest


ArffPropsQueriesTwoTest - Class in test.ml2db.dataharness
:   Class

ArffPropsQueriesTwoTest() - Constructor for class test.ml2db.dataharness.ArffPropsQueriesTwoTest


ArffPropsTableTest - Class in test.ml2db.weka
:   Class

ArffPropsTableTest() - Constructor for class test.ml2db.weka.ArffPropsTableTest


ArffPropsTwoTest - Class in test.ml2db.dataharness
:   Class

ArffPropsTwoTest() - Constructor for class test.ml2db.dataharness.ArffPropsTwoTest


ArffRecordsTable - Class in main.ml2db.weka
:   Class to store the name and column types for the database table which stores
    the ARFF records.

ArffRecordsTable(DbManager) - Constructor for class main.ml2db.weka.ArffRecordsTable


ArffTest - Class in test.ml2db.weka
:   Class

ArffTest() - Constructor for class test.ml2db.weka.ArffTest


ARROW\_STYLE - Static variable in class main.ml2db.mulan.mulanarff.NeighboursPrinter


ATT1 - Static variable in class test.ml2db.dataharness.DataOne


ATT1\_TO\_STRING - Static variable in class test.ml2db.dataharness.DataOne


ATT1\_TO\_STRING - Static variable in class test.ml2db.mulan.mulanarff.MulanArffTest


ATT2 - Static variable in class test.ml2db.dataharness.DataOne


ATT2\_TO\_STRING - Static variable in class test.ml2db.dataharness.DataOne


ATT2\_TO\_STRING - Static variable in class test.ml2db.mulan.mulanarff.MulanArffTest


ATT3 - Static variable in class test.ml2db.dataharness.DataOne


ATT3\_TO\_STRING - Static variable in class test.ml2db.dataharness.DataOne


ATT3\_TO\_STRING - Static variable in class test.ml2db.mulan.mulanarff.MulanArffTest


ATT4 - Static variable in class test.ml2db.dataharness.DataOne


ATT4\_TO\_STRING - Static variable in class test.ml2db.dataharness.DataOne


ATT4\_TO\_STRING - Static variable in class test.ml2db.mulan.mulanarff.MulanArffTest


ATT5 - Static variable in class test.ml2db.dataharness.DataOne


ATT5\_TO\_STRING - Static variable in class test.ml2db.dataharness.DataOne


ATT5\_TO\_STRING - Static variable in class test.ml2db.mulan.mulanarff.MulanArffTest


ATT\_EC\_1111\_TO\_STRING - Static variable in class test.ml2db.mulan.mulanarff.MulanArffTest


ATT\_EC\_111\_TO\_STRING - Static variable in class test.ml2db.mulan.mulanarff.MulanArffTest


ATT\_EC\_11\_TO\_STRING - Static variable in class test.ml2db.mulan.mulanarff.MulanArffTest


ATT\_EC\_1\_TO\_STRING - Static variable in class test.ml2db.mulan.mulanarff.MulanArffTest


ATT\_EC\_2222\_TO\_STRING - Static variable in class test.ml2db.mulan.mulanarff.MulanArffTest


ATT\_EC\_222\_TO\_STRING - Static variable in class test.ml2db.mulan.mulanarff.MulanArffTest


ATT\_EC\_22\_TO\_STRING - Static variable in class test.ml2db.mulan.mulanarff.MulanArffTest


ATT\_EC\_2\_TO\_STRING - Static variable in class test.ml2db.mulan.mulanarff.MulanArffTest


AttFiltArff - Class in main.ml2db.mulan.attributesfiltered


AttFiltArff(String, DbManagers, AttFilter, int) - Constructor for class main.ml2db.mulan.attributesfiltered.AttFiltArff


AttFiltArffTest - Class in test.ml2db.mulan.attributesfilter


AttFiltArffTest() - Constructor for class test.ml2db.mulan.attributesfilter.AttFiltArffTest


AttFiltDataSetGeneratorTest - Class in test.ml2db.mulan.attributesfilter


AttFiltDataSetGeneratorTest() - Constructor for class test.ml2db.mulan.attributesfilter.AttFiltDataSetGeneratorTest


AttFilter - Class in main.ml2db.mulan.attributesfiltered
:   This class filters the attributes of a dataset (the test set) to make it
    compatible with another dataset (the training set).

AttFilter(int, int, DbManagers) - Constructor for class main.ml2db.mulan.attributesfiltered.AttFilter


AttFilterTest - Class in test.ml2db.mulan.attributesfilter


AttFilterTest() - Constructor for class test.ml2db.mulan.attributesfilter.AttFilterTest


ATTRIBUTE\_FIELD - Static variable in class test.ml2db.dataharness.DataTableTest


AttributeFactory - Class in main.ml2db.weka
:   The attribute factory produces binary and numeric Weka attributes.

AttributeFactory(DataSetGenerator) - Constructor for class main.ml2db.weka.AttributeFactory
:   Takes the attribute names from the dataset generator object.

AttributeFactoryTest - Class in test.ml2db.weka
:   Class

AttributeFactoryTest() - Constructor for class test.ml2db.weka.AttributeFactoryTest


attributeIsValid(Attribute) - Static method in class main.ml2db.weka.AttributeFactory
:   Check if an attribute is valid (not null, non-empty name)

AttributesByFrequencyPicker - Class in main.ml2db.al.utility.pickers.byfrequency
:   Class

AttributesByFrequencyPicker() - Constructor for class main.ml2db.al.utility.pickers.byfrequency.AttributesByFrequencyPicker


attributesColumn() - Static method in class test.ml2db.dataharness.DataOne


attributesSection(Instances) - Method in class main.ml2db.weka.DataSetWriter
:   Gets the weka arff string header including all attributes and the "@data"
    label,
    but excluding all instances.

attributesValues() - Static method in class test.ml2db.dataharness.DataOne
:   Results of a select distinct instance, attribute query

AttributeUtils - Class in main.ml2db.mulan.mulanarff


AttributeUtils() - Constructor for class main.ml2db.mulan.mulanarff.AttributeUtils


AttSetByFrequencyPicker - Class in main.ml2db.al.utility.pickers.byfrequency
:   Serves the instances in order of frequency of their attribute sets (most
    frequent first).

AttSetByFrequencyPicker() - Constructor for class main.ml2db.al.utility.pickers.byfrequency.AttSetByFrequencyPicker


AttSetByFrequencyPickerTest - Class in test.ml2db.al.utility.pickers
:   Class

AttSetByFrequencyPickerTest() - Constructor for class test.ml2db.al.utility.pickers.AttSetByFrequencyPickerTest


AUTHOR - Static variable in class main.ml2db.ProjectParameters

A B C D E F G H I J K L M N O P Q R S T U V W X

- Overview
- Package
- Class
- Use
- Tree
- Deprecated
- Index
- Help

- Prev Letter
- Next Letter

- Frames
- No Frames

- All Classes
